# Effectiveness and safety of Hwangryunhaedok-Tang (Huang-Lian-Jie-Du-Tang, Oren-Gedoku-to) for dyslipidemia: A protocol for a PRISMA-compliant systematic review and meta-analysis: Erratum

**DOI:** 10.1097/MD.0000000000024546

**Published:** 2021-01-29

In the article, “Effectiveness and safety of Hwangryunhaedok-Tang (Huang-Lian-Jie-Du-Tang, Oren-Gedoku-to) for dyslipidemia: A protocol for a PRISMA-compliant systematic review and meta-analysis”,^[1]^ the title was first published incorrectly and has since been changed to “Effectiveness and safety of Hwangryunhaedok-Tang (Huang-Lian-Jie-Du-Tang, Oren-Gedoku-to) for dyslipidemia: A PRISMA-compliant systematic review and meta-analysis.”
